# Acquisition of Avian-Origin PB1 Facilitates Viral RNA Synthesis by the 2009 Pandemic H1N1 Virus Polymerase

**DOI:** 10.3390/v12030266

**Published:** 2020-02-28

**Authors:** Fangzheng Wang, Guanqun Liu, Yao Lu, Magda Hlasny, Qiang Liu, Yan Zhou

**Affiliations:** 1 Vaccine and Infectious Disease Organization - International Vaccine Centre (VIDO-InterVac), University of Saskatchewan, Saskatoon, SK S7N 5E3, Canada; fangzheng.wang@usask.ca (F.W.); guanqun.liu@usask.ca (G.L.); yao.lu@usask.ca (Y.L.); mth280@mail.usask.ca (M.H.); qiang.liu@usask.ca (Q.L.); 2 Department of Veterinary Microbiology, Western College of Veterinary Medicine, University of Saskatchewan, Saskatoon, SK S7N 5B4, Canada

**Keywords:** influenza A virus polymerase, avian-origin PB1, 2009 pandemic H1N1 virus

## Abstract

The constant crosstalk between the large avian reservoir of influenza A viruses (IAV) and its mammalian hosts drives viral evolution and facilitates their host switching. Direct adaptation of an avian strain to human or reassortment between avian-origin gene segments with that of human strains are the two mechanisms for the emergence of pandemic viruses. While it was suggested that the 1918 pandemic virus is of avian origin, reassortment of 1918 human isolates and avian influenza viruses led to the generation of 1957 and 1968 pandemic viruses. Interestingly, the avian PB1 segment, which encodes the catalytic subunit of IAV polymerase, is present in the 1957 and 1968 pandemic viruses. The biological consequence and molecular basis of such gene exchange remain less well understood. Using the 2009 pandemic H1N1 virus as a model, whose polymerase contains a human-origin PB1 subunit, we demonstrate that the acquisition of an avian PB1 markedly enhances viral RNA synthesis. This enhancement is also effective in the absence of PB2 adaptive mutations, which are key determinants of host switching. Mechanistically, the avian-origin PB1 does not appear to affect polymerase assembly but imparts the reassorted pandemic polymerase-augmented viral primary transcription and replication. Moreover, compared to the parental pandemic polymerase, the reassorted polymerase displays comparable complementary RNA (cRNA)-stabilizing activity but is specifically enhanced in progeny viral RNA (vRNA) synthesis from cRNA in a trans-activating manner. Overall, our results provide the first insight into the mechanism via which avian-origin PB1 enhances viral RNA synthesis of the 2009 pandemic virus polymerase.

## 1. Introduction

Influenza A virus (IAV) has a broad host range in which the avian species constitute the largest natural reservoir. The spillover of avian strains to mammalian hosts leads to occasional fatal cases and, more frequently, reassortment occurs through the exchange of viral gene segments derived from different origin [1]. The reassortment event not only expands viral diversity but also facilitates host switching and the emergence of novel viruses with pandemic potential. Except for the 1918 pandemic virus whose genesis is under debate [2,3,4,5], all the other three major pandemic viruses (i.e., 1957, 1968, and 2009) arose indisputably as a consequence of genetic reassortment [2,6,7]. These viral strains contain genome segments that are directly contributed by avian viruses or can be traced back to avian origins. Notably, the reassortment process is subjected to constraints, among which the incompatibility of polymerase subunits may lead to a loss of viral fitness [8]. The IAV polymerase is composed of three subunits which act in concert to catalyze viral RNA synthesis. The PB1 subunit is the catalytic core of the polymerase complex, while the PB2 and PA subunits are well characterized for their roles in the cap-snatching process of viral messenger RNA (mRNA) synthesis [9]. Due to stronger functional constraints, PB1 exhibits lower evolutionary rate and limited evolutionary divergence between host-specific lineages [10]. In contrast, PB2 and PA coevolve and encompass the majority of host adaptive mutations, such as the well-known host range determinant at the residue 627 of PB2 [10,11].

It was long observed that human seasonal and pandemic IAV strains, such as those of 1957 and 1968, frequently contain reassorted PB1 segments originated from avian viruses [6,12]. While these reassortment events partially corroborate the genetic compatibility of avian PB1 with human PB2 and PA, the biological consequence and molecular basis of such frequent emergence of avian-origin PB1 remain unclear. In mammalian cells, introducing avian PB1 of H7N1 or H2N2 into the background of mammalian H1N1 and H3N2 polymerases led to increased or comparable activity [13]. An avian H5N1 PB1 also rescued the attenuated polymerase activity and partially restored viral replication of a PB2-627E containing mammalian H1N1 virus [14]. Moreover, the reassortment between a human H3N2 and an avian H5N1 virus led to the generation of an avian PB1-containing reassortant with increased virulence in vivo, although the reassorted polymerase activity remained unchanged in vitro [15]. Swapping the PB1 segment of a human seasonal H2N2 virus with that of the 1968 pandemic strain, which is of avian-origin, increased viral polymerase activity and facilitated replication and transmission of the reassortant virus in guinea pigs [16].

The 2009 pandemic H1N1 (pH1N1) is a “quadruple reassortant” virus resulted from the reassortment between the “triple reassortant” North American swine H1N2 viruses and the Eurasian “avian-like” swine H1N1 viruses. It contains the PB2 and PA gene segments of avian-origin, and the PB1 segment originated from human seasonal H3N2 viruses. Although the seasonal H3N2 PB1 gene is originated from an avian virus, it is circulating in humans since 1968, and PB1 acquired some mammalian-associated mutations [17]. In a ferret model, the 2009 pH1N1 exhibited increased viral replication and pathogenicity compared to a seasonal H1N1 strain [18], whereas it showed mild to moderate virulence in mice compared to the reconstructed 1918 pandemic H1N1 and a highly pathogenic H5N1 virus [19]. Due to the co-circulation of 2009 pandemic viruses with avian strains, it is of significant interest to monitor the potential reassortment events that may further confer the 2009 pH1N1 gene segments of avian origin. Interestingly, upon the reassortment between a 2009 pN1H1 and an avian H9N2, reassortant viruses containing the avian PB1 were mostly more virulent than that containing the pandemic PB1 [20]. However, the underlying mechanism remains unknown.

Here, we further address the biological significance of the acquisition of avian PB1 by the 2009 pH1N1 virus polymerase. We found that the avian-origin PB1 significantly enhances pandemic polymerase activity, and such enhancement is independent of the PB2 adaptive mutations. Mechanistically, the avian-origin PB1 does not affect polymerase assembly but imparts the reassorted pandemic polymerase-augmented viral primary transcription and replication. Moreover, the reassorted polymerase displays comparable complementary RNA (cRNA)-stabilizing activity to the parental polymerase but is specifically enhanced in viral RNA (vRNA) synthesis from cRNA in a trans-activating manner. Overall, our results provide the first insight into the mechanism via which avian-origin PB1 enhances viral RNA synthesis of the 2009 pandemic virus polymerase.

## 2. Materials and Methods

### 2.1. Cells and Viruses

Madin-Darby canine kidney (MDCK) and human embryonic kidney 293T (293T) cells were cultured in minimal essential medium (MEM, Sigma) and Dulbecco’s modified Eagle’s medium (DMEM, Sigma) supplemented with 10% fetal bovine serum (FBS, Gibco, Grand Island, NY, USA) and 50 μg/mL gentamicin, respectively. Chicken embryonic fibroblast (DF-1) cells were cultured in DMEM supplemented with 10% FBS, 4 mM l-Glutamine (Gibco), and 100 U/mL penicillin–streptomycin (Gibco). MDCK cells and 293T cells were maintained at 37 °C; DF-1 cells were grown at 39 °C. A/Halifax/210/2009 (pH1N1) and the recombinant virus pH1N1-PB1_ON_ carrying the PB1 segment of A/turkey/Ontario/6213/1966 (H5N1) were generated by the eight-plasmid reverse-genetics system as previously described [21]. Both viruses were propagated and titrated in MDCK cells, and the correctness of PB1 segments was confirmed by DNA sequencing (Eurofins Genomics, Toronto, ON, Canada).

### 2.2. Virus Growth Curve

A549 cells were infected with the respective virus at a multiplicity of infection (MOI) of 5. The supernatant was harvested at the indicated time; virus titer was then determined by plaque assay.

### 2.3. DNA Constructs and Transfection

Plasmids encoding the polymerase (PB2, PB1, and PA) and NP proteins of a panel of IAV strains were constructed in the pcDNA3.1(−) backbone. These include A/Halifax/210/2009 (pH1N1), A/Turkey/Ontario/6213/1966 (H5N1), A/chicken/British Columbia/CN-6/2004 (H7N3), and A/British Columbia/1/2015 (H7N9). ΔPB1-F2 construct was created by introducing two stop codons (12th and 58th residues) into the +1 open reading frame (ORF) of H5N1 PB1 gene. Site-directed mutagenesis on viral polymerases (PB2-S590G/R591Q, PB2-E627K, and PB1-D445A/D446A) was introduced by overlapping PCR. The human Pol I-driven reporter (pPOLI-vNP-fLuc) and chicken Pol I-driven reporter (pchPOLI-vNA-fLuc) constructs were described elsewhere [22,23]. The plasmids generating full-length vRNA of segment 5 or 6 of pH1N1 (pHH21-pH1N1-vNP and pHH21-pH1N1-vNA) were constructed in the pHH21 backbone [24]. Transient transfection of 293T and DF-1 cells was performed using TransIT-LT1 (Mirus Bio) as per manufacturer’s instructions.

### 2.4. Minireplicon Assay and NP-Free Reconstitution

The minireplicon assay was performed as previously described [23]. Briefly, 293T or DF-1 cells were co-transfected with plasmids (50 ng each) expressing indicated PB2, PB1, PA, and NP proteins together with pPOLI-vNP-fLuc (50 ng) and a constitutively expressed *Renilla* luciferase plasmid pTK-RLuc (50 ng). At 24 h post-transfection (h.p.t), cells were lysed and the relative luciferase activity (RLU) was determined using the Dual-Luciferase Reporter Assay System (Promega, Madison, WI, USA). For NP-free reconstitution, 293T cells were co-transfected with indicated plasmids (100 ng each) expressing PB2, PB1, and PA proteins along with pHH21-pH1N1-vNP generating the full-length segment 5 vRNA. At 48 h.p.t, NP protein expression was determined by immunoblotting.

### 2.5. Split Luciferase Complementation Assay (SLCA)

As previously described [25], the N-terminal (1–229) or C-terminal (230–311) fragment of the *Renilla* luciferase was fused to different termini of full-length PB1 or PA of pH1N1 or H5N1 virus as indicated via a linker (GGGSGGGS). These constructs were designated LN-PB1, PB1-LC, PB1-LN, PA-LC, LN-PA, and LC-PA to indicate the location of the N- or C-terminal portion of the luciferase (LN or LC) relative to polymerase proteins. To select functional SLCA constructs, 293T cells were co-transfected with indicated plasmids expressing PB1 and PA carrying luciferase fragments at different termini (50 ng). The *Renilla* luciferase activity was measured at 24 h.p.t. The LN-PB1 and PA-LC constructs which demonstrated consistent interaction were used for subsequent analysis. Where it is indicated, an inhibitor, R160792 (Sigma), which specifically inhibits PB1 and PA interaction, was added to the media (40 μM) at 5 h.p.t. When indicated, plasmids expressing pH1N1 PB2 (pcDNA-pH1N1-PB2), NP (pcDNA-pH1N1-NP), and segment 6 vRNA (pHH21-pH1N1-vNA) were supplemented to examine PB1–PA interaction in the context of heterotrimeric polymerase complex or vRNP.

### 2.6. cRNA Stabilization Assay

The cRNA stabilization assay was performed as previously described [26]. Briefly, 293T cells were co-transfected with plasmids expressing three polymerase subunits (250 ng each) and NP (1 μg). At 24 h.p.t, cells were infected with the indicated virus at an MOI of 10 in the presence of cycloheximide (CHX, 100 µg/mL). Total RNA was extracted from infected cells at 1 or 6 h post infection (h.p.i) using the TRIzol Reagent (Life Technology, Carlsbad, CA, USA) as per the manufacturer’s instructions. The levels of input vRNA at 1 h.p.i and that of vRNA and cRNA at 6 h.p.i were quantified by a strand-specific real-time RT-PCR.

### 2.7. Strand-Specific Real-Time RT-PCR

The relative quantitation of vRNA and cRNA in the cRNA stabilization assay was determined by a strand-specific real-time RT-PCR as previously described [23,27,28]. Briefly, total RNA (350 ng) was reverse-transcribed at 60 °C for 1 h in a 20-μL reaction containing 10 pmol 5′-tagged RT primer, 0.5 mM dNTP mix, 1 × first-strand buffer, 5 mM DTT, 50 U RNaseOUT, 200 U SuperScript III reverse transcriptase (Invitrogen, Carlsbad, CA, USA), and 32.5% saturated trehalose (Sigma). The RT primers for vRNA and cRNA of pH1N1 segment 6 were 5′–GGCCGTCATGGTGGCGAATGAACACAAGAGTCTGAATGTGC–3′ and 5′–GCTAGCTTCAGCTAGGCATCAGTAGAAACAAGGAGTTTTTTGAAC–3′, respectively. Real-time PCR was performed in a 20-μL mixture containing 2 μL cDNA, 500 nM primers, and 1 × Power SYBR Green PCR master mix (Applied Biosystems, Foster City, CA, USA ) using a StepOnePlus real-time PCR system (Applied Biosystems). The PCR primers for vRNA were 5′–GGCCGTCATGGTGGCGAAT–3′ and 5′– ACTAGAATCAGGATAACAGGAGC–3′ and those for cRNA were 5′–TGTATAAGACCTTGCTTCTGGG–3′ and 5′–GCTAGCTTCAGCTAGGCATC–3′. Relative fold change of vRNA and cRNA levels was normalized to GAPDH mRNA levels (5′–TGCACCACCAACTGCTTAGC–3′ and 5′–GGCATGGACTGTGGTCATGAG–3′) and expressed using the ΔΔCt method relative to the conditions as indicated.

### 2.8. Immunoblotting

Viral NP protein was probed with rabbit antiserum raised in house. β-Actin was probed with a mouse anti-β-actin antibody (CST). Immunoblotting was performed as previously described [23], and nitrocellulose membranes were visualized with an Odyssey infrared imaging system (LI-COR).

### 2.9. Statistical Analysis

The statistical analysis was performed in GraphPad Prism 7 (GraphPad Software, Inc., San Diego, CA, USA) using an unpaired Student’s *t*-test, one-way ANOVA followed by Dunnett test, or two-way ANOVA followed by Sidak test. Data are shown as means ± SD of three independent experiments with samples assayed in triplicate. Significant differences are denoted by *p < 0.05, ** *p* < 0.01, *** *p* < 0.001, or **** *p* < 0.0001.

## 3. Results

### 3.1. Avian-Origin PB1 Enhances 2009 pH1N1 Virus Polymerase Activity

**Figure 1:**
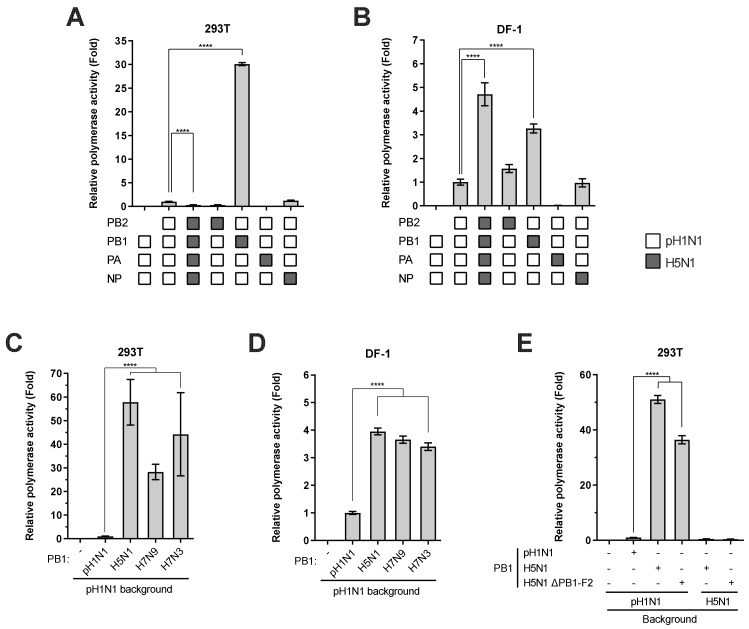
Avian-origin PB1 enhances 2009 pH1N1 virus polymerase activity. (**A** and **B**) 293T (**A**) or DF-1 (**B**) cells were co-transfected with plasmids expressing indicated PB2, PB1, PA, and NP proteins of the pH1N1 and the avian H5N1 virus, along with a human or chicken polymerase I-driven firefly luciferase reporter plasmid. A constitutively expressed *Renilla* luciferase served as an internal control. (**C** and **D**) The PB1 gene of pH1N1 polymerase was replaced by that of avian H5N1, H7N9, or H7N3 in 293T (**C**) or DF-1 (**D**) cells. The omission of PB1 (−PB1) served as a negative control. (**E**) The PB1 gene of pH1N1 or H5N1 polymerase was replaced by a mutant construct deficient in PB1-F2 in 293T cells. The relative luciferase activity was determined at 24 h post transfection (h.p.t) and expressed as fold change relative to the wild-type set of pH1N1 polymerase. Significant differences were determined by one-way ANOVA followed by Dunnett test; **** *p* < 0.0001.

To simulate the potential acquisition of polymerase genes by the 2009 pH1N1 from an avian strain, we replaced individually the PB2, PB1, or PA gene in the pH1N1 background with that derived from an avian virus and monitored the activity of the reassorted polymerases in a minireplicon assay. We chose the low pathogenic A/turkey/Ontario/6213/1966 (H5N1) as the parental avian virus background which is the progenitor of the first confirmed highly pathogenic avian influenza virus in North America [29]. As expected, the parental avian H5N1 polymerase exhibited restricted activity in mammalian 293T cells, but higher activity than the pH1N1 polymerase in chicken DF-1 cells (Figure 1A,B). Replacement of the PB2 gene in the pH1N1 background with the H5N1 counterpart led to reduced and comparable polymerase activity in 293T and DF-1 cells, respectively. Exchange of PA largely abolished the activity of the reassorted polymerases, indicating an incompatibility of the polymerase subunits. Strikingly, acquisition of the H5N1 PB1 subunit by the pH1N1 polymerase markedly enhanced polymerase activity compared to the parental polymerase in 293T cells (Figure 1A). A similar augmentation was observed in DF-1 cells, albeit to a lesser extent (Figure 1B). It is noted that the two PB1 proteins share 96% identify with a 25-amino-acid difference. Consistent with the H5N1 PB1, PB1 genes derived from other avian strains, including a low pathogenic H7N3 virus (A/chicken/British Columbia/CN-6/2004) and a highly pathogenic H7N9 virus (A/British Columbia/1/2015), also significantly enhanced the activity of the reassorted viral polymerases in both 293T (Figure 1C) and DF-1 cells (Figure 1D), ruling out a strain-specific effect. In contrast to that of 2009 pH1N1 [30], PB1 genes of the avian origin mostly encode the viral auxiliary protein PB1-F2 from a +1 open reading frame (ORF), which was shown to indirectly regulate viral polymerase activity through interacting with PB1 [31]. To assess the potential contribution of PB1-F2 derived from avian PB1 genes to the polymerase activity enhancing effect, two stop codons were introduced into the +1 ORF of H5N1 PB1 gene which abolished PB1-F2 expression with the PB1 expression unaffected. In the H5N1 background, PB1-F2 deficiency did not affect H5N1 polymerase activity. The reassorted pH1N1 polymerase containing ΔPB1-F2 H5N1 PB1 was also enhanced in activity to a comparable level as that containing wild-type H5N1 PB1 (Figure 1E). Collectively, these results demonstrated that the avian-origin PB1 enhances 2009 pH1N1 polymerase activity independently of PB1-F2.

### 3.2. Avian-Origin PB1 Enhances 2009 pH1N1 Polymerase Activity Independently of PB2 Adaptive Mutations

**Figure 2:**
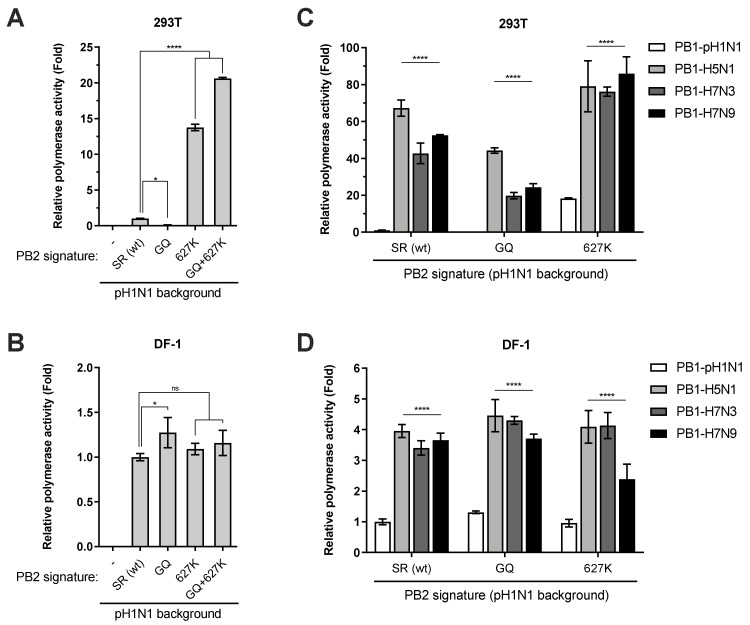
Avian-origin PB1 enhances 2009 pH1N1 polymerase activity independently of PB2 adaptive mutations. (**A** and **B**) 293T (**A**) or DF-1 (**B**) cells were co-transfected with plasmids expressing PB1, PA, and NP proteins of pH1N1 virus along with indicated PB2 carrying S590G/R591Q (GQ), E627K (627K), or GQ+627K mutations.(**C** and **D**) The PB1 gene of pH1N1 polymerase harboring indicated PB2 signatures was replaced by that of avian H5N1, H7N9, or H7N3 in 293T (**C**) or DF-1 (**D**) cells. The relative luciferase activity was determined at 24 h.p.t. and expressed as fold change relative to the wild-type set of pH1N1 polymerase. Significant differences were determined by one-way ANOVA followed by Dunnett test (**A** and **B**) or two-way ANOVA followed by Sidak test (**C** and **D**); * *p* < 0.05, **** *p* < 0.0001, ns, not significant.

The adaptation of avian influenza viruses to a mammalian host, such as human, is usually associated with the accumulation of adaptive mutations within the polymerase genes. A single amino-acid residue at the position 627 of PB2 is a key host range determinant; avian and human viruses mostly contain a glutamic acid (E627) and a lysine (K627) residue, respectively. Unlike previous pandemic viruses, the PB2 subunit of 2009 pH1N1 polymerases contains the avian-like signature E627. Instead, it obtains second-site adaptive mutations, G590S/Q591R (i.e., SR polymorphism), to overcome the restriction of viral polymerase [32]. We were interested in understanding the polymerase-enhancing effect of avian-origin PB1 in relation to PB2 adaptive mutations in the 2009 pH1N1 background. To this end, we firstly validated the activities of pH1N1 polymerases harboring distinct PB2 signatures. Consistent with the previous report, while replacing the SR polymorphism with the avian consensus sequence (i.e., G590Q591, GQ) significantly impaired polymerase activity in 293T cells, a single E627K mutation could dramatically enhance polymerase activity regardless of the 590/591 signatures (Figure 2A). In comparison, all these PB2 variant polymerases displayed comparable activities in avian cells (Figure 2B). 

Next, we introduced avian-origin PB1 of different viral subtypes into pH1N1 polymerases containing distinct PB2 signatures and monitored activities of the reassorted polymerases. Surprisingly, regardless of PB2 590/591 signatures, acquisition of an avian PB1 by the 2009 pH1N1 polymerase variants led to a dramatic enhancement of activity in 293T cells (Figure 2C). Even in the presence of fully adapted PB2 carrying K627, avian-origin PB1 still enhanced polymerase activity by more than four-fold (Figure 2C). The polymerase-enhancing effect was similarly observed in DF-1 cells, albeit to a lesser extent (Figure 2D). Collectively, these results demonstrated that the avian-origin PB1 enhanced 2009 pH1N1 polymerase activity independently of PB2 adaptive mutations. Acquisition of an avian PB1 could fully overcome the restriction rendered by the removal of mammalian PB2 signatures in the pH1N1 background.

### 3.3. Avian-Origin PB1 Has a Minor Effect on Viral Polymerase Complex Assembly

**Figure 3:**
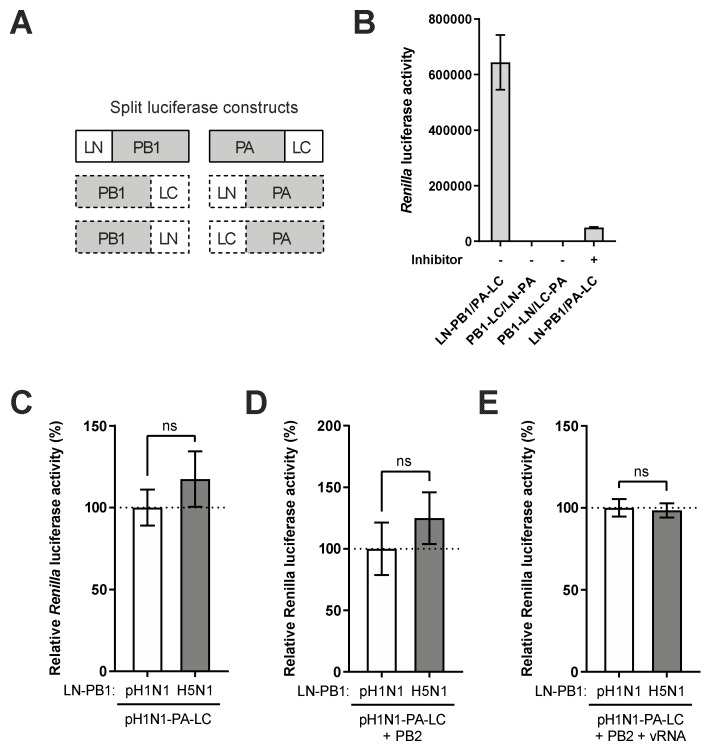
Avian-origin PB1 has a minor effect on viral polymerase complex assembly. (**A**) Schematic diagram of PB1 and PA constructs used in split luciferase complementation assay (SLCA). LN: luciferase N-terminus. LC: luciferase C-terminus. (**B**) 293T cells were co-transfected with indicated SLCA plasmids expressing pH1N1 PB1 and PA with N- or C-terminal tagged *Renilla* luciferase fragments (LN or LC). Where it is indicated, the inhibitor R160792 was added at 5 h.p.t. The *Renilla* luciferase activities were determined at 24 h.p.t. (**C**–**E**) 293T cells were co-transfected with plasmids expressing LN-PB1 of pH1N1 or H5N1 along with PA-LC of pH1N1 (**C**). When indicated, the plasmid expressing PB2 of pH1N1 (**D**) or plasmids expressing PB2 and segment 6 vRNA of pH1N1 (**E**) were supplemented. The *Renilla* luciferase activities were determined at 24 h.p.t and expressed as relative *Renilla* luciferase activity (%) relative to the condition with LN-PB1 and PA-LC of pH1N1. Significant differences were determined by an unpaired Student’s *t*-test; ns, not significant.

To understand the molecular mechanism via which avian-origin PB1 enhanced 2009 pH1N1 polymerase activity, we firstly tested the hypothesis that the avian PB1 may mediate bona fide polymerase assembly more efficiently than the pandemic human PB1. While the newly synthesized PB2 enters the nucleus on its own for polymerase assembly, PB1 and PA subunits were shown to form a heterodimer before nuclear import and interacting with PB2. Thus, we used PB1-PA interaction as an indicator of polymerase assembly and monitored the interaction of PA with avian or pandemic human PB1 using a split luciferase complementation assay. We were able to validate the PB1–PA interaction using pH1N1 constructs in which PB1 and PA were tagged with each half of the *Renilla* luciferase at the N-terminus (LN-PB1) and C-terminus (PA-LC), respectively. The C-terminally tagged PB1 and N-terminally tagged PA showed no interaction regardless we switched the N- or C-terminal half of *Renilla* luciferase gene, indicating an interference with the interacting domains (Figure 3A,B). The inhibitor R160792 that specifically blocks PB1–PA interaction [33] significantly reduced luciferase activity, further validating the assay (Figure 3B). Accordingly, the H5N1 PB1 was tagged at the N-terminus and compared with pH1N1 PB1 for the level of PA interaction. Co-transfection of 293T cells with pH1N1 PA along with H5N1 PB1 showed comparable *Renilla* luciferase activity to that with pH1N1 PB1, suggesting similar levels of protein interaction (Figure 3C). To further monitor such interaction in the context of heterotrimeric polymerase or viral ribonucleoprotein (vRNP) complex, the pH1N1 PB2 and/or NP plus a vRNA-expressing construct were expressed along with PB1 and PA. Under all conditions, the interaction of H5N1 PB1 with pH1N1 PA was similar to that of pH1N1 PB1 with PA (Figure 3D,E). Collectively, these results suggested that avian-origin PB1 did not affect PB1–PA interaction and viral polymerase complex assembly.

### 3.4. Avian-Origin PB1 Enhances Viral Primary Transcription

**Figure 4:**
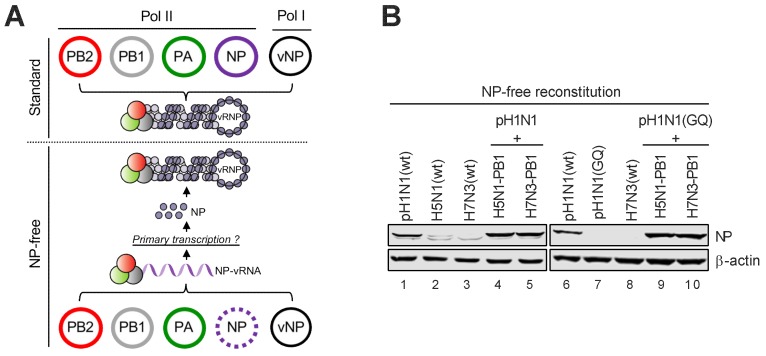
Avian-origin PB1 enhances viral primary transcription. (**A**) Schematic diagram of the NP-free reconstitution system. (**B**) 293T cells were co-transfected with three Pol II plasmids expressing polymerase (PB2, PB1, and PA) of indicated influenza A virus (IAV) strains along with a Pol I plasmid generating the full-length segment 5 viral RNA (vRNA) of pH1N1. The levels of NP protein expression were determined by immunoblotting at 24 h.p.t.

Given that PB1 functions as the catalytic core of the viral polymerase, we next hypothesized that avian PB1 might directly affect the synthesis of viral RNA species. Depending on the mode of viral polymerase, it can act as a transcriptase to catalyze viral mRNA synthesis from viral genomic RNA (vRNA), or a replicase to synthesize more vRNA via the replicative intermediate complementary RNA (cRNA). While the minireplicon assay results as shown in Figure 1 and Figure 2 directly reflected viral mRNA levels, it remained unclear which arm of the viral polymerase activity was enhanced as a result of an avian PB1 acquisition since an enhanced replication activity (i.e., more vRNA) could also lead to more viral mRNA production. To specifically assess whether avian-origin PB1 enhances viral transcriptase activity, we established a modified minireplicon system in which the Pol II-driven NP plasmid was omitted (Figure 4A). Since ongoing viral replication requires concurrent NP encapsidation of newly synthesized vRNA/cRNA, such NP-free condition would largely diminish viral replication and the production of full-length progeny vRNA. In contrast, NP is not required for viral primary transcription; full-length viral mRNA is able to be synthesized from input vRNA followed by translation into viral proteins. Taking advantage of this property, we particularly supplied to the NP-free system a vRNA-expressing plasmid encoding the viral NP segment (Pol I-NP). We rationalized that, if the viral polymerase conducted certain level of primary transcription, the resulting NP mRNA would be translated into NP protein, which could feed back to the NP-free minireplicon system, converting the NP-free condition back to the NP-competent condition. Thus, the accumulation of NP as detected by Western blotting reflects the primary transcription activity of the viral RNA polymerase. This could not be achieved by supplying with Pol-I constructs encoding other segments or luciferase gene. Using this system, we showed that viral polymerases of avian origins (i.e., H5N1 and H7N3) were highly restricted at the level of primary transcription (Figure 4B, lanes 2, 3, and 8). Removal of the SR polymorphism within the PB2 subunit of pH1N1 polymerase also diminished primary transcription (Figure 4B, lane 7 vs. lane 6). Strikingly, under both the SR and GQ backgrounds, introducing an avian-origin PB1 enhanced or restored viral primary transcription (Figure 4B, lanes 4, 5, 9, and 10), echoing the polymerase-enhancing effect observed in Figure 2C. Collectively, these results demonstrated that avian-origin PB1 enhances the transcription activity of the pH1N1 polymerase.

### 3.5. Avian-Origin PB1 Enhances Progeny vRNA Synthesis in Trans

**Figure 5:**
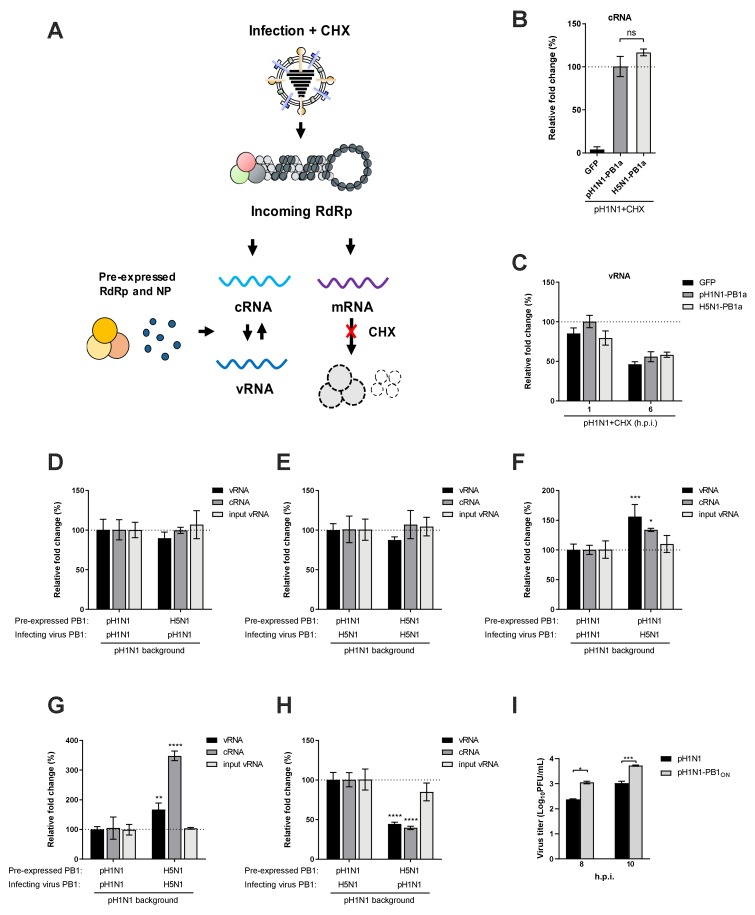
Avian-origin PB1 enhances progeny vRNA synthesis in trans. (**A**) A schematic diagram of the experimental setting. (**B**) 293T cells were co-transfected with plasmids expressing the PB2, PA, and NP of pH1N1 along with a catalytically inactive PB1 (PB1a) of pH1N1 or H5N1. Pre-expression of GFP in place of PB1a served as a negative control. At 24 h.p.t, cells were further infected with the pH1N1 virus at a multiplicity of infection (MOI) of 10 in the presence of cycloheximide (CHX) (100 μg/mL). Total RNA was extracted at 6 h.p.i, and the levels of complementary RNA (cRNA) were determined by strand-specific qPCR. Relative fold change (%) was normalized to GAPDH mRNA levels and expressed using the ΔΔCt method relative to the pH1N1 PB1a condition (set to 100%). (**C**) 293T cells were co-transfected and infected as in (**B**), and the levels of vRNA were determined by strand-specific qPCR at 1 and 6 h.p.i. Relative fold change (%) was expressed relative to the pH1N1 PB1a condition at 1 h.p.i (set to 100%). (**D** and **E**) 293T cells were pre-expressed with PB2, PA, and NP of pH1N1 along with the PB1 of pH1N1 or H5N1 for 24 h. Cells were subsequently infected with the wild-type pH1N1 virus (**D**) or a recombinant pH1N1 virus containing the PB1 segment derived from H5N1 (**E**) at an MOI of 10 in the presence of CHX. (**F**) 293T cells were pre-expressed with the polymerase and NP of pH1N1 for 24 h and subsequently infected with the wild-type pH1N1 or the recombinant pH1N1 virus containing H5N1 PB1 as in (**D** and **E**). (**G** and **H**) 293T cells were pre-expressed with PB2, PA, and NP of pH1N1 along with the PB1 of pH1N1 or H5N1 for 24 h. Cells were subsequently infected with the wild-type pH1N1 or the recombinant pH1N1 virus containing H5N1 PB1 in a homologous (**G**) or a heterologous (**H**) setting relative to the pre-expressed set of polymerases. The levels of input vRNA were determined at 1 h.p.i, and those of cRNA and progeny vRNA were determined at 6 h.p.i. Relative fold change (%) was expressed relative to the pH1N1 PB1 pre-expression condition (set to 100%, **D**–**H**). (**I**) A549 cells were infected with either pH1N1 virus or the pH1N1-PB1_ON_ virus which carries PB1 segment derived from avian origin at an MOI of 5. Virus titer is determined in a single cycle replication setting by plaques assay. Significant differences were determined by one-way ANOVA followed by Dunnett test (**B**) or two-way ANOVA followed by Sidak test (**F**–**I**); * *p* < 0.05, ** *p* < 0.01, *** *p* < 0.001, **** *p* < 0.0001, ns, not significant.

We next asked whether the acquisition of an avian-origin PB1 would augment the replication activity of the pH1N1 polymerase. Influenza virus replication is a two-step process; the incoming vRNP firstly synthesizes the replicative intermediate cRNA, which in turn serves as the template to synthesize progeny vRNA. Given that cRNA stability is a prerequisite for ongoing viral replication, we firstly explored whether the reassorted polymerase containing an avian-origin PB1 has a stronger ability to stabilize nascent cRNA and to prevent it from degradation. We used a cRNA stabilization assay in which 293T cells were pre-expressed with viral polymerase complex and NP before being infected with the pH1N1 virus in the presence of a protein synthesis inhibitor cycloheximide (CHX) [34,35]. The experimental setting is illustrated in Figure 5A. Under this condition, nascent cRNAs synthesized from the incoming vRNPs would be assembled into cRNPs containing the pre-expressed viral protein components to avoid degradation. Moreover, the pre-expressed polymerase complex contained a catalytically inactive PB1 subunit (PB1a) to abrogate cRNP replication, which eliminated interference from accumulative cRNA synthesis. Consistent with previous reports [26], the cRNA-stabilizing effect could only be detected in cells pre-expressing an intact viral polymerase complex but not that with the PB1 subunit replaced by GFP (Figure 5B). Pre-expression of polymerase complexes containing either pH1N1 or H5N1 PB1a led to similar levels of cRNA accumulation as determined by a strand-specific qPCR, indicating comparable cRNA stabilization abilities. It is also noted that input vRNA levels remained consistent across conditions at 1 h post infection (h.p.i.) and 6 h.p.i (Figure 5C), ruling out the possibility of any difference in viral infection.

Given that both the pandemic and avian PB1 conferred comparable cRNA stabilization at the cRNP level, we next sought to examine whether cRNPs containing PB1 of different origin had differential vRNA-synthesizing ability. To this end, the pre-expressed catalytically inactive PB1a in the cRNA stabilization assay was replaced with the active counterpart to resume cRNP replication. Following pH1N1 infection in the presence of CHX, nascent cRNP containing H5N1 PB1 synthesized comparable levels of progeny vRNA to that containing pH1N1 PB1 (Figure 5D), demonstrating that PB1 of both origins, in the context of cRNP, exhibited similar vRNA-synthesizing ability.

To further investigate the polymerase-enhancing effect of avian-origin PB1 in relation to viral RNA replication, we constructed a recombinant pH1N1 virus in which the PB1 segment was replaced with that of H5N1. This recombinant virus showed a one-log increase in replication during a single-cycle infection in A549 cells (Figure 5I). In 293T cells pre-expressed with catalytically inactive PB1a of pH1N1, the recombinant virus synthesized similar levels of cRNA to the parental pH1N1, demonstrating that the avian PB1 as contained in the incoming vRNPs conferred no advantage to cRNA synthesis. Infection of 293T cells pre-expressing active PB1 of pH1N1 or H5N1 with the recombinant virus also showed similar levels of cRNA and progeny vRNA accumulation, corroborating the results that cRNP containing either PB1 had comparable vRNA-synthesizing ability (Figure 5E). Strikingly, when cells pre-expressing pH1N1 PB1 were infected with either virus, the H5N1 PB1-containing recombinant virus led to the synthesis of cRNA and progeny vRNA significantly more than the parental pH1N1 virus (Figure 5F). This indicated that the incoming vRNP-associated PB1 determined in trans the progeny vRNA synthesis by nascent cRNPs. To reinforce this notion, we performed infections in a “homologous” or a “heterologous” fashion in which cells were pre-expressed with the set of polymerases same as or different from that of the infecting virus. Under both settings, the H5N1 PB1-containing recombinant virus gave rise to greater amounts of cRNA and progeny vRNA synthesis, regardless of the pre-expressed sets of polymerases (Figure 5G,H). Collectively, these results demonstrated that the avian-origin PB1 contained in the incoming vRNP enhanced cRNP replication in trans. 

## 4. Discussion

The recurrent presence of avian-origin PB1 in the past pandemic viruses was long observed. By simulating reassortment events between avian and human viruses, previous studies suggested that PB1 genes of human seasonal and 2009 pandemic strains (i.e., human-derived), but not those of avian strains usually show a lack of or attenuated ability to increase viral pathogenicity in mice [20,36]. In this study, we further characterized in vitro the biological significance of the acquisition of an avian PB1 by the 2009 pH1N1 virus, with an emphasis on its effect on viral polymerase activity. Consistent with the effect of avian PB1 on the polymerase activity of human seasonal strains [13,14,16], introducing an avian-origin PB1 into the 2009 pandemic virus background drastically increased the activity of the viral polymerase. The reassorted pandemic polymerase also exhibited enhanced activity when the well-characterized PB2 adaptive mutations were removed, indicating an alternate pathway of mammalian adaptation. Such PB2 independency is reminiscent of replacing the avian PA in the avian polymerase complex by a human PA, which also bypassed avian PB2 restriction in human cells [32]. It is, thus, conceivable that a polymerase constellation containing an avian PB1 and a human PA, such as that of the 1957 and 1968 pandemic viruses and of several experimental reassortants [6,13,15,16], constitutes an additional adaptive strategy to optimize viral polymerase activity in restrictive mammalian hosts. Given that the 2009 pandemic viruses contain a PA segment of avian origin, it would be of importance to examine whether a combined substitution of PB1 and PA with their avian/human counterparts would further enhance polymerase activity or provide any fitness advantage. Interestingly, upon reassortment between an avian H9N2 and a 2009 pH1N1, all H9 reassortants with increased virulence in mice harbored the PA segment from the pandemic virus [20], indicating that the 2009 pandemic PA, albeit of avian origin, already adapted well in mammalian hosts.

In an exploration of the mechanism via which avian-origin PB1 enhanced viral polymerase activity in the 2009 pandemic virus background, we found that it did not appear to affect viral polymerase assembly but promoted viral RNA synthesis from primary transcription and replication. The avian PB1 containing incoming vRNP or cRNP did not directly impact the replication activity of the resident viral polymerase. In contrast, elevated accumulation of viral replication products was observed when incoming vRNP containing an avian PB1 was paired with cRNP in infected cells, indicating that a trans-acting effect on cRNP determined the overall level of viral replication. While a previous report using purified cRNP showed that the cRNP-resident polymerase catalyzed vRNA synthesis in vitro only when purified viral polymerases were provided in trans [35], the source of the trans-acting polymerase during IAV infection remains unknown. In our study, that avian PB1 containing vRNP promoted overall viral replication regardless of the cRNP-resident polymerases indicated that incoming vRNP-associated polymerases serve as a source of trans-acting polymerases. This model is also in line with a previous report that the PB2-627 signature of the incoming vRNP determined the level of host restriction regardless of the PB2-627 signature of cRNP [37]. Although the mechanism of cRNP trans-activation via incoming vRNP warrants further investigation, we propose that a dynamic interaction between the two may act as a molecular switch regulating the process of viral replication. Interestingly, a recent cryo-EM study suggested that transient tetramer formation of polymerase complexes is required for viral replication [38], highlighting a plausible involvement of higher order RNP–RNP interactions.

In summary, our results provide a previously unknown mechanism via which avian-origin PB1 promotes the activity of the 2009 pandemic virus polymerase. Whether the corresponding virus will increase the pathogenicity in vivo remains unknown. The 2009 pH1N1 virus has been circulating in humans for over 10 years and becomes a seasonal virus. In this regard, the pH1N1 virus is beneficial in not acquiring mutations that lead to an increased polymerase activity, such as acquiring the avian-origin PB1 or PB2-627K. A recent study may partially explain the benefit, as PB1 of pH1N1 carrying mammalian-associated glycine at amino acid 216 (PB1-216G) could acquire Oseltamivir-resistant mutation at a faster rate than the virus carrying an avian-associated serine (PB1-216S) [17], which allows the pH1N1 virus to escape from therapeutic treatment. However, monitoring the introduction of avian PB1 genes into the 2009 pandemic influenza viruses is warranted for the emergence of a potential influenza virus with increased pathogenicity.
